# Phospholipase A_2_ Drives Tumorigenesis and Cancer Aggressiveness through Its Interaction with Annexin A1

**DOI:** 10.3390/cells10061472

**Published:** 2021-06-11

**Authors:** Lara Vecchi, Thaise Gonçalves Araújo, Fernanda Van Petten de Vasconcelos Azevedo, Sara Teixeria Soares Mota, Veridiana de Melo Rodrigues Ávila, Matheus Alves Ribeiro, Luiz Ricardo Goulart

**Affiliations:** 1 Laboratory of Nanobiotechnology, Federal University of Uberlandia, Uberlandia 38400-902, MG, Brazil; laravecchi7@yahoo.it (L.V.); tgaraujo@ufu.br (T.G.A.); fvpetten@yahoo.com (F.V.P.d.V.A.); saratsm.s@hotmail.com (S.T.S.M.); 2 Laboratory of Genetics and Biotechnology, Federal University of Uberlandia, Patos de Minas 387400-128, MG, Brazil; matheusribeiromarketing@gmail.com; 3 Laboratory of Biochemistry and Animal Toxins, Institute of Biotechnology, Federal University of Uberlandia, Uberlandia 38400-902, MG, Brazil; veridiana@ufu.br

**Keywords:** lipid metabolism, phospholipase A_2_, Annexin A1, tumorigenesis, tumor microenvironment, cancer aggressiveness

## Abstract

Phospholipids are suggested to drive tumorigenesis through their essential role in inflammation. Phospholipase A_2_ (PLA_2_) is a phospholipid metabolizing enzyme that releases free fatty acids, mostly arachidonic acid, and lysophospholipids, which contribute to the development of the tumor microenvironment (TME), promoting immune evasion, angiogenesis, tumor growth, and invasiveness. The mechanisms mediated by PLA_2_ are not fully understood, especially because an important inhibitory molecule, Annexin A1, is present in the TME but does not exert its action. Here, we will discuss how Annexin A1 in cancer does not inhibit PLA_2_ leading to both pro-inflammatory and pro-tumoral signaling pathways. Moreover, Annexin A1 promotes the release of cancer-derived exosomes, which also lead to the enrichment of PLA_2_ and COX-1 and COX-2 enzymes, contributing to TME formation. In this review, we aim to describe the role of PLA_2_ in the establishment of TME, focusing on cancer-derived exosomes, and modulatory activities of Annexin A1. Unraveling how these proteins interact in the cancer context can reveal new strategies for the treatment of different tumors. We will also describe the possible strategies to inhibit PLA_2_ and the approaches that could be used in order to resume the anti-PLA_2_ function of Annexin A1.

## 1. Introduction

Cancer incidence and mortality have been growing rapidly worldwide. According to the World Health Organization (WHO), in 2020, except for non-melanoma skin cancer, 18 million new cases of the disease and 9.8 million deaths were recorded worldwide, making cancer the second cause of death in the population. The term cancer covers more than 200 diseases, which are histologically and molecularly different. Overall, according to the latest WHO estimate, the most diagnosed tumors in 2020 were: breast (2.26 million cases), lung (2.20 million cases), colorectal (1.93 million cases), prostate (1.41 million), and stomach (1.08 million cases). For the year 2040, an increase of approximately 11.4 million cases (63.4%) is estimated [1]. In the United States, around 1.8 million new cancers will be diagnosed in 2020, with 606,000 deaths [2]. Carcinogenesis is a dynamic process in which transformed cells express different hallmarks during their evolution [3]. Hanahan and Weinberg proposed common and fundamental characteristics for the promotion and progression of tumors, which, despite being a unifying set of organizing principles, must be analyzed as closely related aspects that promote the transformation of normal cells and the stochastic advance of the disease [3,4]. The sustaining proliferative signaling is the main hallmark, once the homeostasis of the cell cycle is interrupted during tumorigenesis. The persistence of non-canonical stimuli, genomic instability, overexpression of receptors, recycling of markers, and epigenetic alterations are events responsible for conferring survival advantages and the selection of clones of malignant cells, which do not follow a fixed path that guarantees a highly heterogeneous environment [5,6].

However, malignant transformation requires the destabilization of multiple signals, which allows not only the survival of tumor cells but also their ability to progress and even to resist therapeutic strategies [7]. The evasion of growth suppression and cell death signals are also crucial in determining these phenotypes with the inactivation of tumor suppressor genes, imbalance in the repair mechanisms, and alterations in the dynamics of the telomeres. Associated with these characteristics, metabolic rewriting immune modulation and abetting microenvironment support continuous cell growth and proliferation. Finally, vascularization and activation of tissue invasion and metastasis establish the ability of these cells to invade surrounding tissue and seed distant sites, which defines an advanced and generally fatal stage of the disease [4,6].

Although the model of clonal evolution and/or selection framework mode proposed by Peter Nowell in 1976 [8] is widely used, it is important to consider that intratumoral heterogeneity also stems from branched evolution and a cooperative environment between different subclones and cell types [7,9]. In this scenario, the molecular make-up of cancer cells is different even at the same tumor site, showing the fundamental role played by the tumor microenvironment (TME) in the initiation and progression of the disease [7]. Abnormal lipid metabolism occurs both in tumor cells and in the TME contributing to the escape from immune system recognition. In this review, we aimed to describe the role of phospholipase A_2_ (PLA_2_), and its interaction with the protein Annexin A1 (AnxA1), in cancer development and progression. We will focus on how such interaction could promote the establishment of an inflammatory and immunosuppressive TME.

## 2. The TME

A tumor is not just an isolated set of molecularly deregulated cells. It is a heterogeneous environment maintained and sustained by dynamism and cooperation between tumor cells at different stages of differentiation and resident host cells, secreted factors, and extracellular matrix (ECM) [10]. TME is a complex and continuously evolving entity that regulates carcinogenesis, controls the initiation, development, and progression of tumors, and impacts therapeutic efficacy. Moreover, the interplay between TME components is mediated by cell-to-cell contact, soluble factors, and extracellular vesicles (EVs) such as exosomes [11]. As proposed by Steven Paget, TME is the fertile “soil” that sustains tumor cells being composed of ECM, fibroblasts, adipocytes, blood vessels, growth factors, hormones, cytokines, and immune cells [10,12]. Extracellular matrix (ECM) represents the non-cellular component of TME, which is secreted by cells and organize in a structure that provides physical support. ECM components are mainly represented by water, polysaccharides, proteoglycans, and proteins such as collagen, fibronectin, and laminin [12,13]. Malignant cells manipulate the ECM by promoting enrichment in matrix remodeling enzymes, such as matrix metalloproteinases (MMPs), and by recruiting cancer-associated fibroblasts (CAFs) [14,15]. This environment established by cancer cells influences the ability of malignant cells to metastasize [16]. Moreover, proteins and transcriptional factors associated with epithelial–mesenchymal transition (EMT) are also activated to reprogram the cellular architecture. Transforming growth factor β (TGF-β) and bone morphogenetic protein (BMP) display essential functions in the EMT switch of tumor cells, stimulating migration and invasion [10].

Blood vessels in TME are responsible to satisfy tumor cell demands, driving angiogenesis, a process by which new blood vessels are developed from a pre-existing vascular network [17]. Hypoxia and the acidic microenvironment trigger this event to restore metabolic equilibrium, coordinated by endothelial cells and regulated by the expression of genes involved in angiogenesis, cell survival, metabolism, and inflammation [3,18]. Vascular endothelial growth factor A (VEGFA) is one of the main mediators of angiogenesis, secreted by tumor cells, together with platelet-derived growth factor (PDGF), and epidermal growth factor (EGF). However, it is the dynamic cooperation present in the TME that sustains the process, involving tumor-associated stromal cells (TASCs), ECM, and leukocytes [17].

The immune cells within TME include neutrophils, macrophages, mast cells, dendritic cells, myeloid-derived suppressor cells, and natural killer cells (innate response), as well as T and B lymphocytes (adaptative response), which may develop tumor-antagonizing or tumor-promoting functions [10,19]. Tumor-infiltrating immune cells represent a prognostic factor in different cancer types, although the complexity of their cellular constituents, cytokines, and mediators determine the course of the disease and even its response to therapy [20]. The TME is characterized for being an immunosuppressive environment where immune regulatory cells and molecules inhibit the activation of T cells [21,22]. TME also presents an abnormal lipid metabolism, with an increase in fatty acid oxidation and a release of eicosanoids from arachidonic acid (AA) [23,24]. Eicosanoids are potent inflammatory mediators that are crucial in the establishment of a highly inflammatory TME, which is a hallmark of cancer. Such lipid metabolism abnormalities confer cancer cells’ resistance to chemotherapy and radiotherapy [24]. Therefore, it is evident that, during tumor promotion and progression, the oxidative damage leads to an imbalance of inflammatory and immunosuppression signaling which results in loss of control of TME.

### Immunosuppressive and Inflammatory Properties of the TME

Although immune cells are essential for tumor control, cancer cells can evade the immune system by modulating markers and signaling pathways [25]. The immunological response relies on the processing of tumoral antigens by antigen-presenting cells (APC) and their subsequent presentation on MHC molecules. APCs are subdivided into dendritic cells (DCs), macrophages, and B cells, being DCs the most potent APCs. In order to get activated, naïve T cells need to receive a co-stimulation through the binding of CD28, expressed on T cells, to B7, which is expressed on APCs. Once activated, T cells proliferate and differentiate into effector T cells. Effector T cells comprise the three types of CD4+ T helper cells (TH), the TH1, TH2, and TH17 types, and the cytotoxic T lymphocytes (CTLs). The distinction between TH1 and TH2 cells is based on the profile of cytokine expression. TH1 response is involved in pathogen clearance and produces IFN-γ, while TH2 cells control parasitic infections and express IL-4, IL-5, and IL-13 [26]. TH17 are pro-inflammatory T cells that produce interleukin 17 (IL-17); they are involved in inflammation, protection against pathogens, and pathogens clearance [27]. It is well known that TH17 cells are actively recruited in the TME of different types of cancer where they can either promote or suppress tumor progression [28,29]. By producing interferon-gamma (IFN-γ), tumor necrosis factor-alpha (TNF-α), and granzymes, CTLs can destroy virus-infected cells and cancer cells [30].

In addition to activating an appropriate T cell profile, an efficient anti-tumor response relies also on the activity of natural killer (NK) cells. They belong to the innate immune system and are specialized in killing cells infected by pathogens. Indeed, NKs express perforins that, by creating holes on the cell membrane, allow the release of granzymes with cytotoxic activity [30,31]. Viral infection and malignant transformation downregulate MHC molecules, and, as a consequence of this event, the cytotoxic activity of NKs is activated [32]. Therefore, NKs expand whether there is an infection or inflammation or whether cancer cells are present.

The TME is an immune suppressive environment characterized by a dysfunction of APCs and by the presence of immune regulatory cells that inhibit T cell priming and suppress CTL function [33,34]. Soluble factors present in TME drive DC tolerization, a process that generates the so-called tolerogenic DCs that lack co-stimulatory molecules on the cell surface and hence, do not activate T cells. In fact, tolerogenic DCs come into contact with T cells inducing their anergy or differentiation into regulatory T cells (Tregs) [35,36]. While Tregs (CD4+ CD25+ Foxp3+) are essential to avoid autoimmunity in normal conditions, in a cancer context they greatly contribute to the immunosuppression in TME [37,38]. In order to exert their regulatory function, Tregs secrete immune suppressive cytokines, such as TGF-β, which inhibit CTL and T cell activation, limiting DC motility [39,40]. TGF-β acts in coordination with chemokines produced by cancer cells and with the local inflammation, recruiting monocytes to the TME and promoting their differentiation into tumor-associated macrophages (TAMs) [41,42]. Macrophages, in turn, can be classified into M1 and M2 macrophages [43]. The signaling through Toll-like receptors (TLRs) and IFN-γ induces the macrophages M1, also called classically activated macrophages. They stimulate immune response and produce pro-inflammatory cytokines. On the contrary, the M2 macrophages, or alternatively activated macrophages, are immunosuppressive being induced by IL-4 and IL-13 and express IL-10. It has been reported that TAMs usually display an M2 phenotype, promoting angiogenesis [43]. TAMs are important mediators of the inflammation in the TME [44] together with tumor-associated stromal cells (TASCs). The main cellular component of the stroma of solid tumors is represented by cancer-associated fibroblasts (CAFs) that cooperate with TAMs to supply the TME with inflammatory mediators and to aid the recruitment of inflammatory cells [45,46]. Moreover, it has been demonstrated that CAFs, TAMs, and APCs in the TME promote the differentiation and expansion of the inflammatory TH17 cells [47,48]. Once in the TME, TH17 cells could eventually differentiate into Tregs [49].

Myeloid-derived suppressive cells (MDCSs) represent another class of cells that are likely recruited by TH17 lymphocytes and activated by the inflammatory microenvironment of TME [50,51]. MDSCs are a group of myeloid progenitors and immature mononuclear cells comparable to monocytes and immature polymorphonucleates [52] that regulate the immune system in both physiological and pathological conditions. Their expansion is particularly enhanced in the presence of cancer cells, inflammation, or infection [53]. MDSCs exert their immunosuppressive function due to the production of IL-10 and TGF-β and due to their activity in inducing Tregs and T cell anergy [54,55]. To summarize, in TME APCs display a reduced antigen presentation function, resulting in decreased T cell activation. In addition, cancer cells do not express the co-stimulatory molecule, B7, contributing to T cell anergy [56]. Finally, certain types of cancer cells express high levels of the protein “programmed death ligand-1” (PDL-1), which displays a suppressive function. Indeed, when PDL-1 binds to its receptor PD1, expressed on T cells, it elicits a signaling cascade that results in T cell inactivation, reduced T cell proliferation, and reduced apoptosis of Tregs [57,58]. However, the cancer-immune evasion is also mediated by several soluble factors and molecules released into EVs, present in TME. In this scenario, PLA_2_ seems to play an essential role in tumorigenesis, cancer progression, and immunosuppression. In particular, the release of AA-induced by PLA_2_ supplies the TME with the inflammatory prostaglandin E2 (PGE2) whose activity is crucial in enhancing inflammation, and immunosuppression [23]. Moreover, changes in the behavior of the endogenous PLA_2_ inhibitor, the protein Annexin A1 (AnxA1), contribute to cancer progression [59]. In particular, the loss of the anti-PLA_2_ activity of AnxA1 could represent a hallmark of cancer aggressiveness.

## 3. Phospholipases: Classification and General Properties

Phospholipases (PLs) are a ubiquitous group of enzymes that share the property of hydrolyzing phospholipids, which are essential components of cell membranes [60]. In nature, phospholipases are widespread and play different roles such as signal transduction, production of lipid mediators and second messengers, digestion of metabolites in humans, and different pharmacological actions in snake venoms. These enzymes vary considerably in structure, function, regulation, and mode of action [61]. The majority of cells contain large amounts of phospholipases that can exist as secreted forms, associated with the membrane or located intracellularly [62]. Thus, PLs belong to classes of hydrolases that catalyze the hydrolysis of ester bonds and phosphate esters in phospholipids, predominantly on glycerophospholipids, also degrading neutral lipids [60]. Depending on where the hydrolytic cleavage of the phospholipid molecule is stimulated by the enzyme, PLs are divided into four classes: PLA (PLA_1_ and PLA_2_), PLB, PLC, and PLD. The phospholipase A_1_ (PLA_1_) and phospholipase A_2_ (PLA_2_) are acyl-hydrolases that are responsible for removing fatty acids from the glycerol structure of the target phospholipid. PLA_1_ also generates a 2-acyl lysophospholipid, by acting on the sn-1 position, while PLA_2_ acts on the sn-2 position releasing a 1-acyl lysophospholipid. The phospholipase B (PLB) hydrolyzes both of these acyl groups and frequently displays also lysophospholipase activity, thus removing the remaining acyl portion in lysophospholipids. In addition to this, some fungal PLBs also exert transacylase activity, thus leading to phospholipid generation from free fatty acids and lysophospholipids. Finally, phospholipase C (PLC) and phospholipase D (PLD) are phosphodiesterases [61,63].

**Figure 1:**
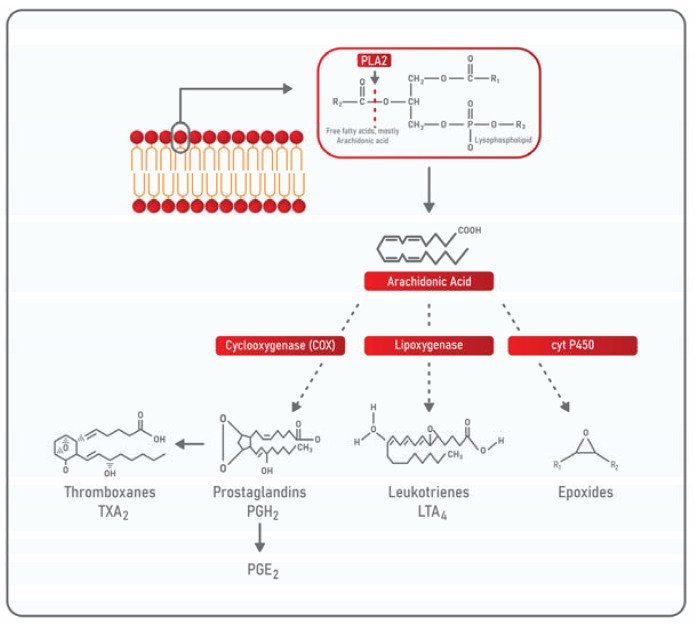
Schematic representation of PLA_2_ cascade. PLA_2_ acts on membrane phospholipids catalyzing the hydrolysis of acyl-ester bonds at the sn-2 position. Upon this hydrolysis, free fatty acids, mainly arachidonic acid (AA), are released. AA is therefore metabolized by COX enzymes to release PGH2, the precursor of thromboxanes and of the highly inflammatory PGE2. AA is also metabolized by lipoxygenase to produce leukotrienes, another class of inflammatory molecules, or by cytochrome P450 to release epoxides.

Phospholipases A_2_ (PLA_2_ EC 3.1.1.4) belong to a PLA superfamily of enzymes, widely distributed in living organisms. PLA_2_s catalyze the hydrolysis of acyl-ester bonds at the sn-2 position of the phospholipids present in the cell membrane [64,65]. The hydrolysis reaction of membrane phospholipids depends on calcium ions, on the catalytic unit of PLA_2_, which is formed by the amino acids Histidine at position 48 and Aspartic acid at position 49, and on a water molecule. The products generated by the catalysis of these enzymes are, on one hand, polyunsaturated fatty acids, mostly AA [66], and, on the other hand, lysophospholipids. In this context, PLA_2_ is involved in determining the phospholipid composition of membranes, in supporting a balance between saturated and unsaturated fatty acids, and in generating epidermal lipid barriers [67,68]. In addition to this, PLA_2_ exerts an important function in producing energy thanks to the release of fatty acids that enter the β-oxidation metabolic pathway [69]. Lysophospholipids function as extracellular mediators, elicit specific G-protein-coupled receptors’ signaling pathways, which are involved in Ca^+2^ homeostasis and various cellular processes, such as proliferation, survival, migration, and adhesion [70]. In this manner, lysophospholipids contribute to different biological processes such as regulation of the immune system, inflammation, and cancer. An important lysophospholipid generated by the action of PLA2 is lysophosphatidylcholine (LPC) [71] that increases cancer metastases [72]. Importantly, lysophospholipids can be further processed by Autotaxin, a PLD enzyme that cleaves the serine/choline groups from lysophosphatidylcholine and lysophosphatidylserine releasing lysophosphatidic acid (LPA) [73]. LPA functions as a mitogen activating the G protein-coupled receptors, LPAR1-6. LPA signaling is frequently dysregulated in cancer being responsible for oncogenesis, cancer cells’ proliferation, and metastasis formation [74]. Once released by the action of PLA_2_, AA is metabolized by various enzymes and participates in the synthesis of eicosanoids such as prostaglandins, prostacyclins, and leukotrienes. Through the action of the enzymes cyclooxygenases (COXs), AA is transformed into the prostaglandin H2 (PGH2) that is the precursor of the highly inflammatory, PGE2, and of thromboxanes that display vasoconstrictor activities [75,76]. Moreover, AA can be oxidized by the enzyme 5-lipoxygenase (LOX) to produce leukotrienes, an additional class of inflammatory mediators. In addition, when AA is metabolized by cytochrome P450, epoxides are produced and act in lowering blood pressure (Figure 1) [77].

The PLA_2_ superfamily comprises several highly different proteins that can be distributed throughout six major classes: cytosolic (cPLA_2_), secreted (sPLA_2_), calcium-independent (iPLA_2_), platelet activator acetyl-hydrolase (PAF-AH), lysosomal (LPLA_2_), and adipose tissue-specific PLA_2_s (AdPLA). Each member of this superfamily has been implicated in lipid metabolism and the first two classes are highly expressed in tumor cells [78,79].

cPLA2 hydrolyzes mainly glycerophospholipids including phosphatidylcholines, phosphatidylethanolamines, and or AA in the sn-2 position, it has a catalytic effect dependent on Ca^2+^ and its high molecular mass can vary between 61 and 114 kDa [66]. These enzymes are widely distributed in most types of human tissue and are responsible for different disturbances including allergic responses and inflammatory damage induced in lung and brain cancer models [80].

The sPLA_2_ group was the first type of PLA_2_ discovered. These enzymes are found in animal venoms, synovial fluid, and various mammalian tissues. sPLA_2_s are classified into 18 main groups (IA, IB, IIA, IIB, IIC, IID, IIE, IIF, III, V, IX, X, XIA, XIB, XIIA, XIIB, XIII, and XIV) and various subgroups according to the homology of sequence [78]. sPLA_2_s have been described as carcinogenic mediators due to the metabolic activity of their reaction products, in particular eicosanoids. These eicosanoids are directly involved in proliferation, survival, differentiation, and inflammation, besides contributing to the establishment and maintenance of important stages of tumor growth and metastasis [81,82]. In addition, it is known that the catalytic activity of PLA_2_s also leads to the production of the platelet-activating factor (PAF), characterized as an important mediator of the inflammatory process during platelet aggregation [83]. In addition to the catalytic role of PLA_2_ in releasing AA, it has been demonstrated that sPLA_2_ displays non-catalytic functions. Indeed, sPLA_2_ can activate membrane receptors expressed on tumor cells triggering intracellular responses that promote cell growth, proliferation, and resistance to metabolic stress and apoptosis. Therefore, understanding the role of sPLA_2_ in the molecular biology of cancer may contribute substantially to the development of additional strategies to control different tumors [81,84].

### PLA_2_ in TME

PLA_2_ regulates lipid metabolism by releasing AA from membrane phospholipids and by promoting the synthesis of eicosanoids [85,86]. In fact, the importance of PLA_2_ in cancer has been described with much effort devoted to depicting the role of sPLA_2_. Different isoforms of sPLA_2_ exist and, among them, sPLA_2_-IIA is upregulated in the lung, prostate, colon, gastric, and breast cancers [87]. sPLA_2_-IIA favors tumorigenesis, proliferation, cell survival, and increases the local inflammation, angiogenesis [81]. This isoform supports the cancer stem cell (CSC) phenotype of lung and prostate cancer cells [88] and correlates with the aggressive castration-resistant prostate cancer (CRPC) [89]. sPLA_2_-IIA has also been found to play an essential role in TME of prostate and lung cancer [90,91]. Increased levels of sPLA_2_ in the TME of prostate cancer patients correlates with a poor prognosis [87,89]. Regarding the isoform sPLA_2_ IID, Miki and collaborators demonstrated that this enzyme acts as an immunosuppressive molecule in skin cancer by increasing the polarization of macrophages towards the M2 phenotype and by diminishing CTL activity [92].

cPLA_2_ and iPLA_2_ also display important roles in cancer. In particular, the expression of cPLA_2_ has been correlated with a worse prognosis in several types of cancer [93] and with angiogenesis in colorectal cancer [94]. Moreover, CRPC expresses higher levels of cPLA_2_ [95]. According to Weiser-Evans and collaborators, deletion of cPLA_2_ alters the TME in such a way that progression of lung cancer is inhibited through macrophage modulation [96]. Regarding iPLA_2_, some studies demonstrated its involvement in ovarian cancer [97,98] and a pro-tumoral role of extracellular and exosome-free iPLA_2_ and cPLA_2_ [99].

In addition to these findings, PLA_2_ acts indirectly as an immunosuppressive molecule through the synthesis of PGE2 and LPA. PGE2 is a highly immunosuppressive molecule, which is significantly expressed in colon, lung, breast, and head and neck cancers [100]. It has been described that PGE2 acts inhibiting NK cells and promoting the expansion of regulatory cells [101,102]. Indeed, it has been demonstrated that PGE2 is one of the major inducers of tolerogenic DCs [103,104] and of MDSC that inhibit the anti-tumor response [51,105]. In addition to this, PGE2 enhances the proliferation and function of Tregs by inducing the expression of the transcription factor, FOXP3, whose activity is necessary for the development of the immunosuppressive functions of Tregs [106]. Moreover, by increasing IL-17 expression, PGE2 promotes the recruitment of macrophages in TME and stimulates their polarization towards the M2 phenotype [107]. On the other side, although little is known about the immunosuppressive activities of LPA, it seems that this molecule can inhibit the anti-tumor effector functions of CTLs [108] and that, its signaling pathway through LPAR supports TAM development [109].

## 4. Annexin A1: An Endogenous PLA_2_ Inhibitor

It is well accepted that PLA_2_ is one of the major players in the establishment of an inflammatory environment and, consequently, it is crucially involved in tumorigenesis and tumor progression [81]. On the other side, a key mediator of the anti-inflammatory response is the 37 kDa protein Annexin A1 (AnxA1). AnxA1 is a phospholipid-binding protein expressed in many tissues and cell types including leukocytes, lymphocytes, endothelial and epithelial cells [110]. AnxA1 is one of the mediators of the anti-inflammatory activity of glucocorticoids (GCs) [111], and exerts its anti-inflammatory activities by inhibiting PLA_2_ in the cytoplasm [112]. AnxA1 also regulates different processes including membrane trafficking, proliferation, differentiation, and apoptosis [113,114].

AnxA1 can be found in its 37 kDa intact form that displays an anti-PLA_2_ activity and in two cleaved forms of 33 and 36 kDa. The 33 kDa cleaved form was described to be pro-inflammatory [115,116] whereas the 36 kDa cleaved form was associated with monocyte recruitment and prevention of inflammation [116]. AnxA1 cleavage is due to elastases, metalloproteases, or proteinases and leads to the release of the AnxA1 N-terminal biological active peptide [117] that signals through the “Formyl peptide receptors” (FPRs) [118]. FPRs are Gi protein-coupled receptors involved in the chemotaxis of leukocytes towards the bacterial chemotactic peptide N-formyl-methionyl-leucyl-phenylalanine (fMLF) [119]. By binding to FPRs, N-formylated peptides elicit a signal cascade involving PI3K and MAPK [120,121]. The anti-inflammatory activity of AnxA1 is exerted by binding to the FPR2 that is expressed on fibroblasts, endothelial cells, stromal cells, and is highly abundant in leukocytes [120,122]. The anti-inflammatory activity of the N-terminal peptide of AnxA1 is, however, 20-fold less potent, a fact that raised the hypothesis that such a peptide could display the role of limiting the action of the intact form of AnxA1 [123]. Nevertheless, AnxA1 has a pro-inflammatory role in certain circumstances. In fact, once phosphorylated by PKC, AnxA1 migrates to nuclei where it induces the expression of pro-inflammatory cytokines [124]. Moreover, the 33 kDa cleaved form of AnxA1 generated by Calpain 1 increases the immobilization of neutrophils on endothelial cells thus facilitating the trans-endothelial migration and promoting inflammation [115]. Finally, it has been shown that in cells infected by influenza virus A the N-terminal peptide of AnxA1 enhances the inflammatory response by activating FPR2 [125].

**Figure 2:**
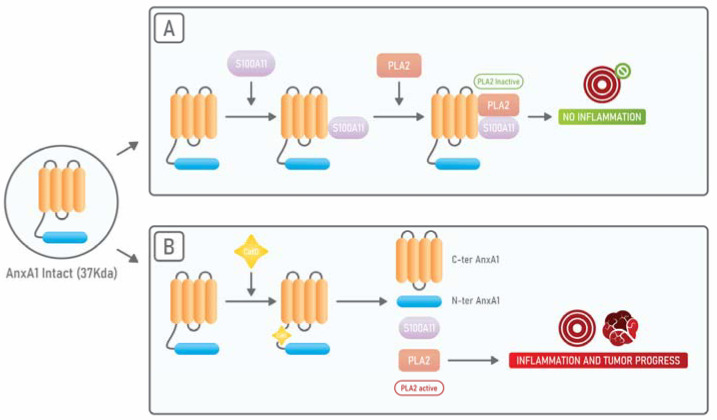
Schematic representation of AnxA1 cleavage and subsequent lack of inhibition of PLA_2_**.** (**A**) When AnxA1 is present in its intact form of 37 kDa, it binds to S100A11 and this complex is able to bind and inhibit PLA2, resulting in inflammation prevention and/or resolution. (**B**) When AnxA1 is cleaved by the protease Cathepsin D (CatD), the release of its N-terminal peptide and its 33 kDa C-terminal portion takes place. Once cleaved in this way, AnxA1 is no longer able to bind to S100A11. The disruption of such complex results in the inability to bind and inhibit PLA_2_. Hence, PLA_2_ remains functional and promotes inflammation and tumor progression.

AnxA1 binds, through its N-terminal portion, to S100A11 another calcium-binding protein and, when AnxA1 is bound to S100A11, it displays a high affinity for cPLA_2_. On the other side, it has been shown that, in squamous carcinoma cells, when there is an exposure to EGF, EGFR phosphorylates AnxA1 in its tyrosine 21 residue and subsequently AnxA1 suffers a proteolytic cleavage at its tryptophan 12 residue by Cathepsin D. Such cleavage results in the dissociation from S100A11 and cPLA_2_. In this way, the anti-cPLA_2_ activity of AnxA1 is abolished and cPLA_2_ can promote tumor growth (Figure 2) [59].

### AnxA1 in TME

AnxA1 has been described as an essential player in several aspects of cancer, such as proliferation, chemoresistance, invasion, and metastasis formation [126]. In fact, AnxA1 plays important roles in the progression of several types of tumors, including astrocitomas, glioblastomas, melanomas, and those affecting the lung, breast, and pancreas [127,128]. In breast cancer, it has been described that AnxA1 supports the metastatic process by promoting the TGF-β/Smad signaling and the subsequent EMT [129]. Interestingly AnxA1 can be found in a secreted form in breast, prostate, and pancreatic cancers. This secreted form of AnxA1 elicits an autocrine signaling cascade through FPR1 that stimulates migration and invasion properties of these tumors [130,131]. The pivotal role of FPR1 signaling has also been described for astrocytomas [132] and neuroblastomas [121].

Recently, AnxA1 has been described to play immunosuppressive roles in the cancer context. It has been shown that AnxA1 promotes the polarization of macrophages towards the M2 phenotype and induces the expression of IL-10 thus facilitating breast cancer progression and metastasis [133,134]. In hepatocellular carcinoma (HCC), it has been shown that the AnxA1 N-terminal peptide is responsible for the polarization of macrophages towards the M2 phenotype, by signaling through FPR2 and by eliciting the activation of ERK, Akt, and NFkB [135]. Indeed, a previous study had shown that a deficiency in FPR2 sustains an M1 phenotype in HCC [136]. It was also reported that AnxA1 plays an essential role in the induction of Tregs in the TME of triple-negative breast cancer models [137].

## 5. PLA_2_ and Annexin A1 in Cancer-Derived Extracellular Vesicles

EVs are lipid bilayer delimited particles that are released from cells. They can be found in different biological fluids regulating inflammation and tissue repair [138], and modulating the immune response, viral pathogenicity, and cancer progression [139,140]. EVs promote intercellular communication through contacting membranes of target cells or by transferring EVs’ cargos, which can be lipids, proteins, and nucleic acids [141]. EVs are classified into endosomal-derived exosomes and plasma membrane-derived MVs, Exosomes originate from multivesicular bodies (MVBs) which are endosomes that contain intraluminal vesicles (ILVs). When MVBs fuse with the plasmatic membrane ILVs are released as exosomes [139,140]. Cancer cells produce copious amounts of both MVs and exosomes that can be found in all biological fluids altering the phenotype of cells with which they come in contact, promoting a pro-tumoral gene expression [142,143]. Tumor-derived exosomes (TDEs) are involved in the increased proliferation and chemoresistance of cancer cells [144], angiogenesis [145], and metastasis [146]. Moreover, the immunosuppressive roles of exosomes have been described. In this scenario, exosomes induce T cell apoptosis [147], decrease DC differentiation [148], and suppress NK cytotoxic response [149]. MVs have also been linked with several pro-tumoral functions such as proliferation, angiogenesis, metastasis, chemoresistance, and immunomodulation [142]. It has been shown that MVs, due to the presence of TGF-β on their surface, can interact with immune cells inducing NK and T cell suppression [150].

**Figure 3:**
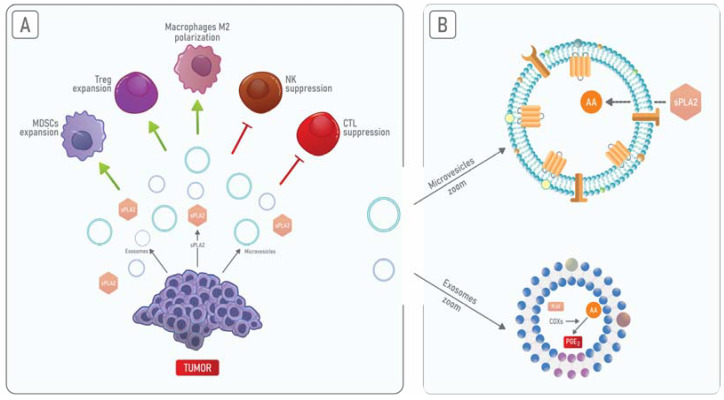
Schematic representation of PLA_2_ and AnxA1 immunosuppressive functions in TME. (**A**) Tumors release AnxA1 cleaved form in microvesicles and either free sPLA_2_ or PLA_2_ localized within tumor-derived exosomes. Such type of release promotes immunosuppression by inhibiting NK and CTL activity while enhancing Tregs and MDSCs expansion and M2 polarization of macrophages. (**B**) Free sPLA_2_ acts on lipids of microvesicles, releasing arachidonic acid (AA). The presence of the AnxA1 cleaved form in microvesicles probably supports sPLA2 action.

sPLA_2_ is present in the extracellular milieu either within exosomes or as an exosome-free secreted form [151]. It has been shown that both of these PLA_2_ forms act on phospholipids present on MVs promoting the release of AA and therefore amplifying the inflammatory process [152,153]. Hence, although exosomes and MVs represent distinct structures, these EVs can cooperate in the induction of the immunosuppression and inflammation of TME (Figure 3).

It has been found that TDEs contain cPLA_2_, iPLA_2_, sPLA_2_, COX-1, and COX-2. Moreover, TDEs are enriched in free fatty acids, including AA, and the immunosuppressive molecule PGE2 [151]. TEDs-associated PGE_2_ promotes tumorigenesis by increasing the expression of cell death protein-ligand (PDL-1), a molecule responsible for immune escape [154]. In breast cancer, TEDs-associated PGE2 is responsible for the release of pro-inflammatory cytokines that leads to the accumulation of MDSC in TME [155,156]. Regarding AnxA1, it is released within EVs [157,158]. Recently, AnxA1 has started to be considered a specific marker of MVs [159] that is localized on the surface of these structures. However, the mechanism through which this happens remains unresolved [160]. Calcium promotes the interaction of AnxA1 with cellular membranes [161] from which it may be loaded on budding MVs [160]. AnxA1 present in EVs promotes the activation of keratinocytes through the activation of FPRs in an autocrine loop [162], which promotes cancer cell motility [157]. Leoni and collaborators also demonstrated that the inhibition of FPR1 and FPR2 abrogated the pro-healing effect of AnxA1 containing EVs [158]. Moreover, EVs containing AnxA1 secreted by prostate epithelial cells may contribute to the suppression of the immune response into the male tract [163]. Finally, AnxA1 has been also described as being essential for the release of exosomes from MVBs [164,165]. Therefore, AnxA1 can promote the release of PLA_2_-enriched exosomes from cancer cells, leading to increased PGE2 levels and subsequently to an increase in the inflammatory response and immunosuppression in TME.

## 6. Use of PLA_2_ Inhibitors to Control Cancer Progression

The use of non-steroidal anti-inflammatory drugs (NSAIDs) or COX-2 inhibitors (COXIBs) has been widely explored during the last years as cancer prevention and treatment strategies. Studies have shown either a decrease incidence of cancer in chronic users or a decrease in mortality rates in cancer patients treated with these drugs [166,167]. Despite that COXIBs display the advantage of non-inducing toxicity in the gastrointestinal tract, their clinical long-term use is limited by their significant cardiotoxicity. Since such side effect is due to a shunting towards leukotriene production [168], growing evidence has shown that the dual inhibition of COX and LOX enzymes would be an efficient and safer option compared to COXIBs alone [169]. In addition to this, the anti-inflammatory activity of corticosteroids drugs is mediated by AnxA1. Therefore, AnxA1 action could be limited in TME due to the presence of its cleaved and pro-inflammatory form. In fact, AnxA1 cleavage could explain why corticosteroids can display a pro-tumoral or an anti-tumoral effect depending on the type of cancer [170]. However, AnxA1 regulates the functions of PLA_2_. Hence, PLA_2_ inhibitors are also interesting as anti-cancer therapeutic strategies. Indeed, sPLA_2_ inhibition, besides avoiding the release of AA and the subsequent production of PGE2, could also interfere with the non-catalytic activities of PLA_2_ in signal transduction pathways that support tumor growth and progression.

Recently, it has been suggested that PGE2 enhances cancer cells’ invasion by increasing the expression of adhesion molecules, such as intercellular adhesion molecule-1 (ICAM-1), and increasing the phosphorylation of anti-apoptotic transcription factor “signal transducer and activator of transcription” (STAT-3). Authors achieved a significant reduction in PGE2 and ICAM-1 expression levels, as well as a reduction in STAT-3 phosphorylation levels by inhibiting sPLA_2_ in lung cancer cells [171,172]. Similarly, the treatment of human esophageal adenocarcinoma cells with sPLA_2_ inhibitor attenuates the expression of ICAM-1 [173] and decreases viability and proliferation of this type of cancer cell [174]. Therefore, the use of molecules and drugs able to inhibit PLA_2_ and especially with anti sPLA_2_ activities could represent a good strategy in order to improve cancer patients’ outcomes.

Varespladib (LY315920) and its orally available form methyl-Varespladib (LY333013) are promising PLA_2_ inhibitors. Those compounds have been developed by the pharmaceutical industry to treat inflammatory diseases. Both are potent and selective inhibitors of the human sPLA_2_ and their inhibitory activity on sPLA_2_ occurs at nano-and picomolar concentrations. Interestingly, these drugs display inhibitory activity against sPLA_2_s present in 28 snake venoms [175,176]. In animal models, Varespladib and methyl-Varespladib have been shown to inhibit atherogenesis since they demonstrated to significantly decrease total cholesterol and to reduce aneurysm formation [177,178]. However, for the treatment of patients with acute coronary syndrome and other inflammatory diseases, such as sepsis and rheumatoid arthritis, these inhibitors failed to show efficacy [176,179].

Another synthetic sPLA_2_ inhibitor is the molecule S3319 that, in lung cancer cells, decreases ICAM-1 expression levels and, subsequently, reduces cancer cell invasion [171]. Different studies indicated that sPLA_2_ could be used as a biomarker and as an important therapeutic target in prostate cancer [180]. The sPLA_2_ mRNA levels were 22-fold overexpressed in prostate cancer cells when compared to normal cells. Two inhibitors of sPLA_2_, cFLSYR, and c(2Nap)LS(2Nap)R, proved to be efficient in attenuating the proliferation of sPLA_2_-positive LNCaP and PC-3 cell lines but not the sPLA_2_-negative DU145 cell line. Curiously, sPLA_2_ is overexpressed in androgen-independent prostate cancer PC3 cells when compared to the androgen-dependent LNCaP cell lines. Therefore, PLA_2_ inhibitors could be used as alternative strategies in the treatment of prostatic tumors and diagnosis of prostate cancer. The use of sPLA_2_ inhibitors could be of particular interest for those prostate cancers that are positive for sPLA_2_ and are non-responsive to androgen due to their androgen-independency.

A natural inhibitor of sPLA_2_-IIA, ochnaflavone, has been shown to strongly inhibit sPLA_2_-IIA activity and, as a result, it could be an interesting molecule in the treatment of inflammatory diseases and cancer [181]. In human aortic smooth muscle cells, the treatment with ochnaflavone inhibited DNA synthesis, and downregulated cyclins and cyclin-dependent kinases (CDKs) thus leading to G1-phase cell cycle arrest [182,183]. Another potent inhibitor of PLA_2_ is the marine natural product scalaradial that showed cytotoxic activity on various cancer cell lines, specifically against HepG2, MCF-7, HeLa, and HCT-116 cells [184].

Maslinic acid, a natural pentacyclic triterpenoid, was proved to inhibit the sPLA_2_ enzyme activity in a concentration-dependent manner. In addition, maslinic acid inhibits the inflammation induced by sPLA_2_, including PGE2 production and differentiation and migration of inflammatory cells [185,186]. Indeed, maslinic acid induces different anti-cancer effects in multiple tumors like those affecting the breast, prostate, pancreas, kidneys, lungs, and gastro-intestinal tract [187,188].

Sulforaphane, a natural isothiocyanate present in cruciferous vegetables, also showed to potently inhibit the expression and activity of sPLA_2_. It exhibited chemoprevention properties and therapeutic potential against several types of cancer including oral, prostate, breast, colon, skin, and urinary bladder cancers. In breast cancer, this bioactive compound has been studied extensively as an anti-cancer agent inhibiting the expression of anti-apoptotic genes and inducing G2-M cell cycle arrest by stabilizing microtubules [175].

Several PLA_2_ inhibitory proteins were purified from the plasma of different species of snakes and are classified into alpha (α), beta (β), and gamma (γ) types, according to their structural features. Thereupon, Gimenes et. al. (2017) demonstrated that γCdcPLI, a sPLA_2_ inhibitor from *Crotalus durissus* collilineatus, has anti-tumor, antimetastatic, and anti-angiogenic properties in MDA-MB-231 breast cancer cells [189]. This inhibitor modulates important mediators of the apoptotic pathway and reduces the production of vascular endothelial growth factor (VEGF) [189].

We already pointed that AnxA1 is an endogenous PLA_2_ inhibitor that can be cleaved by different proteases, including elastase, calpain, plasmin, proteinase 3, and Cathepsin D. Of particular interest is the cleavage of AnxA1 by the soluble lysosomal aspartic endopeptidase (EC 3.4.23.5), Cathepsin D, which is found to be highly expressed in various types of cancers and correlated with metastasis [190]. Cathepsin D cleaves AnxA1 at Trp^12^ residue and unlocks its binding to S100A11, which is essential in order to bind and inhibit PLA_2_. Therefore, upon this cleavage AnxA1 is no longer able to inhibit PLA_2_ [59]. By using the inhibitor of Cathepsin D, Pepstatin A, it was possible to inhibit the amount of cleaved AnxA1, to induce apoptosis, and to decrease the invasion and migration of triple-negative breast cancer cells. These anti-tumorigenic effects were, at least in part, due to resumed inhibition of PLA_2_ by the intact form of AnxA1 [191].

## 7. Final Considerations

The activity of PLA_2_ in TME plays crucial roles in tumor development and progression. PLA_2_ can be found in TME either in a free form that acts on lipids present in MVs or within exosomes. An endogenous inhibitor of PLA_2_ is the anti-inflammatory protein, AnxA1. However, once cleaved by proteases, AnxA1 is no longer able to inhibit PLA_2_ and in this way, it promotes tumor progression. Interestingly, AnxA1 can be found in MVs in its cleaved form that probably supports the action of PLA_2_ on MVs’ lipids. Therefore, the direct inhibition of PLA_2_ or the inhibition of AnxA1 cleavage, with the subsequent resumed anti-PLA_2_ activity, could represent interesting therapeutic strategies in the cancer context. The knowledge presented in this review emphasizes the importance of validating these strategies in order to open the possibility of designing innovative approaches to improve cancer patients’ outcomes.

## Data Availability

No new data were created or analyzed in this study. Data sharing is not applicable to this article.

## References

[1] World Health Organization (2018). Breast Cancer: Prevention and Control. https://www.who.int/cancer/detection/breastcancer/en/.

[2] Siegel R.L., Miller K.D., Jemal A. (2020). Cancer statistics, 2020. CA Cancer J. Clin..

[3] Fouad Y.A., Aanei C. (2017). Revisiting the hallmarks of cancer. Am. J. Cancer Res..

[4] Hanahan D., Weinberg R.A. (2011). Hallmarks of cancer: The next generation. Cell.

[5] Biswas S., Rao C.M. (2017). Epigenetics in cancer: Fundamentals and Beyond. Pharm. Ther..

[6] Vogelstein B., Papadopoulos N., Velculescu V.E., Zhou S., Diaz L.A., Kinzler K.W. (2013). Cancer genome landscapes. Science.

[7] Cleary A.S., Leonard T.L., Gestl S.A., Gunther E.J. (2014). Tumour cell heterogeneity maintained by cooperating subclones in Wnt-driven mammary cancers. Nature.

[8] Nowell P.C. (1976). The clonal evolution of tumor cell populations. Science.

[9] Baca S.C., Prandi D., Lawrence M.S., Mosquera J.M., Romanel A., Drier Y., Park K., Kitabayashi N., MacDonald T.Y., Ghandi M. (2013). Punctuated evolution of prostate cancer genomes. Cell.

[10] Anderson N.M., Simon M.C. (2020). The tumor microenvironment. Curr. Biol..

[11] Garcia-Gomez A., Rodríguez-Ubreva J., Ballestar E. (2018). Epigenetic interplay between immune, stromal and cancer cells in the tumor microenvironment. Clin. Immunol..

[12] Wu T., Dai Y. (2017). Tumor microenvironment and therapeutic response. Cancer Lett..

[13] Mohan V., Das A., Sagi I. (2020). Emerging roles of ECM remodeling processes in cancer. Semin. Cancer Biol..

[14] Erdogan B., Webb D.J. (2017). Cancer-associated fibroblasts modulate growth factor signaling and extracellular matrix remodeling to regulate tumor metastasis. Biochem. Soc. Trans..

[15] Rice A.J., Cortes E., Lachowski D., Cheung B.C.H., Karim S.A., Morton J.P., Del Rio Hernandez A. (2017). Matrix stiffness induces epithelial-mesenchymal transition and promotes chemoresistance in pancreatic cancer cells. Oncogenesis.

[16] Lambert A.W., Pattabiraman D.R., Weinberg R.A. (2017). Emerging Biological Principles of Metastasis. Cell.

[17] De Palma M., Biziato D., Petrova T.V. (2017). Microenvironmental regulation of tumour angiogenesis. Nat. Rev. Cancer.

[18] Carmeliet P., Jain R.K. (2000). Angiogenesis in cancer and other diseases. Nature.

[19] Lei X., Lei Y., Li J.-K., Du W.-X., Li R.-G., Yang J., Li J., Li F., Tan H.-B. (2020). Immune cells within the tumor microenvironment: Biological functions and roles in cancer immunotherapy. Cancer Lett..

[20] Galli F., Aguilera J.V., Palermo B., Markovic S.N., Nistico P., Signore A. (2020). Relevance of immune cell and tumor microenvironment imaging in the new era of immunotherapy. J. Exp. Clin. Cancer Res..

[21] Zhang Y., Ertl H.C. (2016). Starved and Asphyxiated: How Can CD8(+) T Cells within a Tumor Microenvironment Prevent Tumor Progression. Front. Immunol..

[22] Topalian S.L., Drake C.G., Pardoll D.M. (2015). Immune checkpoint blockade: A common denominator approach to cancer therapy. Cancer Cell.

[23] Johnson A.M., Kleczko E.K., Nemenoff R.A. (2020). Eicosanoids in Cancer: New Roles in Immunoregulation. Front. Pharm..

[24] Corn K.C., Windham M.A., Rafat M. (2020). Lipids in the tumor microenvironment: From cancer progression to treatment. Prog. Lipid Res..

[25] Tang S., Ning Q., Yang L., Mo Z., Tang S. (2020). Mechanisms of immune escape in the cancer immune cycle. Int. Immunopharmacol..

[26] Bretscher P. (2019). On Analyzing How the Th1/Th2 Phenotype of an Immune Response Is Determined: Classical Observations Must Not Be Ignored. Front. Immunol..

[27] Zambrano-Zaragoza J.F., Romo-Martinez E.J., Duran-Avelar Mde J., Garcia-Magallanes N., Vibanco-Perez N. (2014). Th17 cells in autoimmune and infectious diseases. Int. J. Inflam..

[28] Kryczek I., Wei S., Szeliga W., Vatan L., Zou W. (2009). Endogenous IL-17 contributes to reduced tumor growth and metastasis. Blood.

[29] Zou W., Restifo N.P. (2010). T(H)17 cells in tumour immunity and immunotherapy. Nat. Rev. Immunol..

[30] Williams M.A., Bevan M.J. (2007). Effector and memory CTL differentiation. Annu. Rev. Immunol..

[31] Jost S., Altfeld M. (2013). Control of human viral infections by natural killer cells. Annu. Rev. Immunol..

[32] Vivier E., Raulet D.H., Moretta A., Caligiuri M.A., Zitvogel L., Lanier L.L., Yokoyama W.M., Ugolini S. (2011). Innate or adaptive immunity? The example of natural killer cells. Science.

[33] Vander Heiden M.G., Cantley L.C., Thompson C.B. (2009). Understanding the Warburg effect: The metabolic requirements of cell proliferation. Science.

[34] Luo Y., Zheng S.G. (2016). Hall of Fame among Pro-inflammatory Cytokines: Interleukin-6 Gene and Its Transcriptional Regulation Mechanisms. Front. Immunol..

[35] Gabrilovich D. (2004). Mechanisms and functional significance of tumour-induced dendritic-cell defects. Nat. Rev. Immunol..

[36] Melief C.J. (2008). Cancer immunotherapy by dendritic cells. Immunity.

[37] Budhu S., Schaer D.A., Li Y., Toledo-Crow R., Panageas K., Yang X., Zhong H., Houghton A.N., Silverstein S.C., Merghoub T. (2017). Blockade of surface-bound TGF-beta on regulatory T cells abrogates suppression of effector T cell function in the tumor microenvironment. Sci. Signal..

[38] Nishikawa H., Sakaguchi S. (2010). Regulatory T cells in tumor immunity. Int. J. Cancer.

[39] Ito T., Wang Y.H., Duramad O., Hanabuchi S., Perng O.A., Gilliet M., Qin F.X., Liu Y.J. (2006). OX40 ligand shuts down IL-10-producing regulatory T cells. Proc. Natl. Acad. Sci. USA.

[40] Weber F., Byrne S.N., Le S., Brown A., Breit S.N., Scolyer R.A., Halliday G.M. (2005). Transforming growth factor-beta1 immobilises dendritic cells within skin tumours and facilitates tumour escape from the immune system. Cancer Immunol. Immunother..

[41] Ruffell B., Affara N.I., Coussens L.M. (2012). Differential macrophage programming in the tumor microenvironment. Trends Immunol..

[42] Chen J.J., Lin Y.C., Yao P.L., Yuan A., Chen H.Y., Shun C.T., Tsai M.F., Chen C.H., Yang P.C. (2005). Tumor-associated macrophages: The double-edged sword in cancer progression. J. Clin. Oncol..

[43] Sica A., Allavena P., Mantovani A. (2008). Cancer related inflammation: The macrophage connection. Cancer Lett..

[44] Mantovani A., Schioppa T., Porta C., Allavena P., Sica A. (2006). Role of tumor-associated macrophages in tumor progression and invasion. Cancer Metastasis. Rev..

[45] Schoppmann S.F., Birner P., Stockl J., Kalt R., Ullrich R., Caucig C., Kriehuber E., Nagy K., Alitalo K., Kerjaschki D. (2002). Tumor-associated macrophages express lymphatic endothelial growth factors and are related to peritumoral lymphangiogenesis. Am. J. Pathol..

[46] Iijima J., Konno K., Itano N. (2011). Inflammatory alterations of the extracellular matrix in the tumor microenvironment. Cancers.

[47] Kryczek I., Banerjee M., Cheng P., Vatan L., Szeliga W., Wei S., Huang E., Finlayson E., Simeone D., Welling T.H. (2009). Phenotype, distribution, generation, and functional and clinical relevance of Th17 cells in the human tumor environments. Blood.

[48] Miyahara Y., Odunsi K., Chen W., Peng G., Matsuzaki J., Wang R.F. (2008). Generation and regulation of human CD4+ IL-17-producing T cells in ovarian cancer. Proc. Natl. Acad. Sci. USA.

[49] Guery L., Hugues S. (2015). Th17 Cell Plasticity and Functions in Cancer Immunity. BioMed Res. Int..

[50] Veglia F., Perego M., Gabrilovich D. (2018). Myeloid-derived suppressor cells coming of age. Nat. Immunol..

[51] Bunt S.K., Sinha P., Clements V.K., Leips J., Ostrand-Rosenberg S. (2006). Inflammation induces myeloid-derived suppressor cells that facilitate tumor progression. J. Immunol..

[52] Marvel D., Gabrilovich D.I. (2015). Myeloid-derived suppressor cells in the tumor microenvironment: Expect the unexpected. J. Clin. Investig..

[53] Gabrilovich D.I., Nagaraj S. (2009). Myeloid-derived suppressor cells as regulators of the immune system. Nat. Rev. Immunol..

[54] Lindau D., Gielen P., Kroesen M., Wesseling P., Adema G.J. (2013). The immunosuppressive tumour network: Myeloid-derived suppressor cells, regulatory T cells and natural killer T cells. Immunology.

[55] Huang B., Pan P.Y., Li Q., Sato A.I., Levy D.E., Bromberg J., Divino C.M., Chen S.H. (2006). Gr-1+CD115+ immature myeloid suppressor cells mediate the development of tumor-induced T regulatory cells and T-cell anergy in tumor-bearing host. Cancer Res..

[56] Zippelius A., Batard P., Rubio-Godoy V., Bioley G., Lienard D., Lejeune F., Rimoldi D., Guillaume P., Meidenbauer N., Mackensen A. (2004). Effector function of human tumor-specific CD8 T cells in melanoma lesions: A state of local functional tolerance. Cancer Res..

[57] Chemnitz J.M., Parry R.V., Nichols K.E., June C.H., Riley J.L. (2004). SHP-1 and SHP-2 associate with immunoreceptor tyrosine-based switch motif of programmed death 1 upon primary human T cell stimulation, but only receptor ligation prevents T cell activation. J. Immunol..

[58] Driessens G., Kline J., Gajewski T.F. (2009). Costimulatory and coinhibitory receptors in anti-tumor immunity. Immunol. Rev..

[59] Sakaguchi M., Murata H., Sonegawa H., Sakaguchi Y., Futami J., Kitazoe M., Yamada H., Huh N.H. (2007). Truncation of annexin A1 is a regulatory lever for linking epidermal growth factor signaling with cytosolic phospholipase A2 in normal and malignant squamous epithelial cells. J. Biol. Chem..

[60] Dennis E.A. (2015). Introduction to Thematic Review Series: Phospholipases: Central Role in Lipid Signaling and Disease. J. Lipid Res..

[61] Wilton D.C., Vance D.E., Vance J.E. (2008). CHAPTER 11—Phospholipases. Biochemistry of Lipids, Lipoproteins and Membranes.

[62] Aloulou A., Rahier R., Arhab Y., Noiriel A., Abousalham A. (2018). Phospholipases: An Overview. Methods Mol. Biol..

[63] Brown W.J., Chambers K., Doody A. (2003). Phospholipase A2 (PLA2) enzymes in membrane trafficking: Mediators of membrane shape and function. Traffic.

[64] Azevedo F.V., Lopes D.S., Cirilo Gimenes S.N., Ache D.C., Vecchi L., Alves P.T., Guimaraes Dde O., Rodrigues R.S., Goulart L.R., Rodrigues V.d.M. (2016). Human breast cancer cell death induced by BnSP-6, a Lys-49 PLA(2) homologue from Bothrops pauloensis venom. Int. J. Biol. Macromol..

[65] de Vasconcelos Azevedo F.V.P., Zoia M.A.P., Lopes D.S., Gimenes S.N., Vecchi L., Alves P.T., Rodrigues R.S., Silva A.C.A., Yoneyama K.A.G., Goulart L.R. (2019). Antitumor and antimetastatic effects of PLA2-BthTX-II from Bothrops jararacussu venom on human breast cancer cells. Int. J. Biol. Macromol..

[66] Burke J.E., Dennis E.A. (2009). Phospholipase A2 biochemistry. Cardiovasc. Drugs Ther..

[67] Ilic D., Bollinger J.M., Gelb M., Mauro T.M. (2014). sPLA2 and the epidermal barrier. Biochim. Biophys. Acta.

[68] Murakami M., Sato H., Taketomi Y. (2020). Updating Phospholipase A2 Biology. Biomolecules.

[69] Slatter D.A., Aldrovandi M., O’Connor A., Allen S.M., Brasher C.J., Murphy R.C., Mecklemann S., Ravi S., Darley-Usmar V., O’Donnell V.B. (2016). Mapping the Human Platelet Lipidome Reveals Cytosolic Phospholipase A2 as a Regulator of Mitochondrial Bioenergetics during Activation. Cell Metab..

[70] Ishii I., Fukushima N., Ye X., Chun J. (2004). Lysophospholipid receptors: Signaling and biology. Annu. Rev. Biochem..

[71] Meyer zu Heringdorf D., Jakobs K.H. (2007). Lysophospholipid receptors: Signalling, pharmacology and regulation by lysophospholipid metabolism. Biochim. Biophys. Acta.

[72] Law S.H., Chan M.L., Marathe G.K., Parveen F., Chen C.H., Ke L.Y. (2019). An Updated Review of Lysophosphatidylcholine Metabolism in Human Diseases. Int. J. Mol. Sci..

[73] Tokumura A., Majima E., Kariya Y., Tominaga K., Kogure K., Yasuda K., Fukuzawa K. (2002). Identification of human plasma lysophospholipase D, a lysophosphatidic acid-producing enzyme, as autotaxin, a multifunctional phosphodiesterase. J. Biol. Chem..

[74] Benesch M.G., Ko Y.M., McMullen T.P., Brindley D.N. (2014). Autotaxin in the crosshairs: Taking aim at cancer and other inflammatory conditions. FEBS Lett..

[75] Smith W.L., DeWitt D.L., Garavito R.M. (2000). Cyclooxygenases: Structural, cellular, and molecular biology. Annu. Rev. Biochem..

[76] Smith W.L., Urade Y., Jakobsson P.J. (2011). Enzymes of the cyclooxygenase pathways of prostanoid biosynthesis. Chem. Rev..

[77] Wang B., Wu L., Chen J., Dong L., Chen C., Wen Z., Hu J., Fleming I., Wang D.W. (2021). Metabolism pathways of arachidonic acids: Mechanisms and potential therapeutic targets. Signal. Transduct. Target. Ther..

[78] Six D.A., Dennis E.A. (2000). The expanding superfamily of phospholipase A(2) enzymes: Classification and characterization. Biochim. Biophys. Acta.

[79] Schaloske R.H., Dennis E.A. (2006). The phospholipase A2 superfamily and its group numbering system. Biochim. Biophys. Acta.

[80] Leslie C.C. (2015). Cytosolic phospholipase A(2): Physiological function and role in disease. J. Lipid Res..

[81] Brglez V., Lambeau G., Petan T. (2014). Secreted phospholipases A2 in cancer: Diverse mechanisms of action. Biochimie.

[82] Cummings B.S., McHowat J., Schnellmann R.G. (2000). Phospholipase A(2)s in cell injury and death. J. Pharmacol. Exp. Ther..

[83] Valentin E., Lambeau G. (2000). Increasing molecular diversity of secreted phospholipases A(2) and their receptors and binding proteins. Biochim. Biophys. Acta.

[84] Hunter K.W., Crawford N.P., Alsarraj J. (2008). Mechanisms of metastasis. Breast Cancer Res. BCR.

[85] Scott K.F., Sajinovic M., Hein J., Nixdorf S., Galettis P., Liauw W., de Souza P., Dong Q., Graham G.G., Russell P.J. (2010). Emerging roles for phospholipase A2 enzymes in cancer. Biochimie.

[86] Peng Z., Chang Y., Fan J., Ji W., Su C. (2021). Phospholipase A2 superfamily in cancer. Cancer Lett..

[87] Jiang J., Neubauer B.L., Graff J.R., Chedid M., Thomas J.E., Roehm N.W., Zhang S., Eckert G.J., Koch M.O., Eble J.N. (2002). Expression of group IIA secretory phospholipase A2 is elevated in prostatic intraepithelial neoplasia and adenocarcinoma. Am. J. Pathol..

[88] Lu S., Dong Z. (2017). Overexpression of secretory phospholipase A2-IIa supports cancer stem cell phenotype via HER/ERBB-elicited signaling in lung and prostate cancer cells. Int. J. Oncol..

[89] Sved P., Scott K.F., McLeod D., King N.J., Singh J., Tsatralis T., Nikolov B., Boulas J., Nallan L., Gelb M.H. (2004). Oncogenic action of secreted phospholipase A2 in prostate cancer. Cancer Res..

[90] Dong Y., Lu B., Zhang X., Zhang J., Lai L., Li D., Wu Y., Song Y., Luo J., Pang X. (2010). Cucurbitacin E, a tetracyclic triterpenes compound from Chinese medicine, inhibits tumor angiogenesis through VEGFR2-mediated Jak2-STAT3 signaling pathway. Carcinogenesis.

[91] Oleksowicz L., Liu Y., Bracken R.B., Gaitonde K., Burke B., Succop P., Levin L., Dong Z., Lu S. (2012). Secretory phospholipase A2-IIa is a target gene of the HER/HER2-elicited pathway and a potential plasma biomarker for poor prognosis of prostate cancer. Prostate.

[92] Miki Y., Kidoguchi Y., Sato M., Taketomi Y., Taya C., Muramatsu K., Gelb M.H., Yamamoto K., Murakami M. (2016). Dual Roles of Group IID Phospholipase A2 in Inflammation and Cancer. J. Biol. Chem..

[93] Caiazza F., McCarthy N.S., Young L., Hill A.D., Harvey B.J., Thomas W. (2011). Cytosolic phospholipase A2-alpha expression in breast cancer is associated with EGFR expression and correlates with an adverse prognosis in luminal tumours. Br. J. Cancer.

[94] Wendum D., Svrcek M., Rigau V., Boelle P.Y., Sebbagh N., Parc R., Masliah J., Trugnan G., Flejou J.F. (2003). COX-2, inflammatory secreted PLA2, and cytoplasmic PLA2 protein expression in small bowel adenocarcinomas compared with colorectal adenocarcinomas. Mod. Pathol..

[95] Patel M.I., Singh J., Niknami M., Kurek C., Yao M., Lu S., Maclean F., King N.J., Gelb M.H., Scott K.F. (2008). Cytosolic phospholipase A2-alpha: A potential therapeutic target for prostate cancer. Clin. Cancer Res..

[96] Weiser-Evans M.C., Wang X.Q., Amin J., Van Putten V., Choudhary R., Winn R.A., Scheinman R., Simpson P., Geraci M.W., Nemenoff R.A. (2009). Depletion of cytosolic phospholipase A2 in bone marrow-derived macrophages protects against lung cancer progression and metastasis. Cancer Res..

[97] Li H., Zhao Z., Wei G., Yan L., Wang D., Zhang H., Sandusky G.E., Turk J., Xu Y. (2010). Group VIA phospholipase A2 in both host and tumor cells is involved in ovarian cancer development. FASEB J..

[98] Xu Y., Xiao Y.J., Zhu K., Baudhuin L.M., Lu J., Hong G., Kim K.S., Cristina K.L., Song L., Williams F.S. (2003). Unfolding the pathophysiological role of bioactive lysophospholipids. Endocr. Metabol. Disord..

[99] Cai Q., Zhao Z., Antalis C., Yan L., Del Priore G., Hamed A.H., Stehman F.B., Schilder J.M., Xu Y. (2012). Elevated and secreted phospholipase A(2) activities as new potential therapeutic targets in human epithelial ovarian cancer. FASEB J..

[100] Wang D., Dubois R.N. (2010). Eicosanoids and cancer. Nat. Rev. Cancer.

[101] Rodriguez-Vita J., Lawrence T. (2010). The resolution of inflammation and cancer. Cytokine Growth Factor Rev..

[102] Holt D.M., Ma X., Kundu N., Collin P.D., Fulton A.M. (2012). Modulation of host natural killer cell functions in breast cancer via prostaglandin E2 receptors EP2 and EP4. J. Immunother..

[103] Korkaya H., Liu S., Wicha M.S. (2011). Breast cancer stem cells, cytokine networks, and the tumor microenvironment. J. Clin. Investig..

[104] Liu Z., Li Z., Mao K., Zou J., Wang Y., Tao Z., Lin G., Tian L., Ji Y., Wu X. (2009). Dec2 promotes Th2 cell differentiation by enhancing IL-2R signaling. J. Immunol..

[105] Sinha P., Clements V.K., Fulton A.M., Ostrand-Rosenberg S. (2007). Prostaglandin E2 promotes tumor progression by inducing myeloid-derived suppressor cells. Cancer Res..

[106] Baratelli F., Lin Y., Zhu L., Yang S.C., Heuze-Vourc’h N., Zeng G., Reckamp K., Dohadwala M., Sharma S., Dubinett S.M. (2005). Prostaglandin E2 induces FOXP3 gene expression and T regulatory cell function in human CD4+ T cells. J. Immunol..

[107] Liu L., Ge D., Ma L., Mei J., Liu S., Zhang Q., Ren F., Liao H., Pu Q., Wang T. (2012). Interleukin-17 and prostaglandin E2 are involved in formation of an M2 macrophage-dominant microenvironment in lung cancer. J. Thorac. Oncol..

[108] Mathew D., Kremer K.N., Strauch P., Tigyi G., Pelanda R., Torres R.M. (2019). LPA5 Is an Inhibitory Receptor That Suppresses CD8 T-Cell Cytotoxic Function via Disruption of Early TCR Signaling. Front. Immunol..

[109] Feng Y., Xiao M., Zhang Z., Cui R., Jiang X., Wang S., Bai H., Liu C., Zhang Z. (2020). Potential interaction between lysophosphatidic acid and tumor-associated macrophages in ovarian carcinoma. J. Inflamm..

[110] Kamal A.M., Flower R.J., Perretti M. (2005). An overview of the effects of annexin 1 on cells involved in the inflammatory process. Mem. Inst. Oswaldo. Cruz..

[111] Yang Y.H., Morand E., Leech M. (2013). Annexin A1: Potential for glucocorticoid sparing in RA. Nat. Rev. Rheumatol..

[112] Sheikh M.H., Solito E. (2018). Annexin A1: Uncovering the Many Talents of an Old Protein. Int. J. Mol. Sci..

[113] Barbosa C.M.V., Fock R.A., Hastreiter A.A., Reutelingsperger C., Perretti M., Paredes-Gamero E.J., Farsky S.H.P. (2019). Extracellular annexin-A1 promotes myeloid/granulocytic differentiation of hematopoietic stem/progenitor cells via the Ca(2+)/MAPK signalling transduction pathway. Cell Death Discov..

[114] Bizzarro V., Fontanella B., Franceschelli S., Pirozzi M., Christian H., Parente L., Petrella A. (2010). Role of Annexin A1 in mouse myoblast cell differentiation. J. Cell Physiol..

[115] Williams S.L., Milne I.R., Bagley C.J., Gamble J.R., Vadas M.A., Pitson S.M., Khew-Goodall Y. (2010). A proinflammatory role for proteolytically cleaved annexin A1 in neutrophil transendothelial migration. J. Immunol..

[116] Blume K.E., Soeroes S., Keppeler H., Stevanovic S., Kretschmer D., Rautenberg M., Wesselborg S., Lauber K. (2012). Cleavage of annexin A1 by ADAM10 during secondary necrosis generates a monocytic “find-me” signal. J. Immunol..

[117] Rescher U., Goebeler V., Wilbers A., Gerke V. (2006). Proteolytic cleavage of annexin 1 by human leukocyte elastase. Biochim. Biophys. Acta.

[118] Gavins F.N., Yona S., Kamal A.M., Flower R.J., Perretti M. (2003). Leukocyte antiadhesive actions of annexin 1: ALXR- and FPR-related anti-inflammatory mechanisms. Blood.

[119] Le Y., Murphy P.M., Wang J.M. (2002). Formyl-peptide receptors revisited. Trends Immunol..

[120] Cattaneo F., Parisi M., Ammendola R. (2013). Distinct signaling cascades elicited by different formyl peptide receptor 2 (FPR2) agonists. Int. J. Mol. Sci..

[121] Snapkov I., Oqvist C.O., Figenschau Y., Kogner P., Johnsen J.I., Sveinbjornsson B. (2016). The role of formyl peptide receptor 1 (FPR1) in neuroblastoma tumorigenesis. BMC Cancer.

[122] Dufton N., Hannon R., Brancaleone V., Dalli J., Patel H.B., Gray M., D’Acquisto F., Buckingham J.C., Perretti M., Flower R.J. (2010). Anti-inflammatory role of the murine formyl-peptide receptor 2: Ligand-specific effects on leukocyte responses and experimental inflammation. J. Immunol..

[123] Purvis G.S.D., Solito E., Thiemermann C. (2019). Annexin-A1: Therapeutic Potential in Microvascular Disease. Front. Immunol..

[124] Zhao B., Wang J., Liu L., Li X., Liu S., Xia Q., Shi J. (2016). Annexin A1 translocates to nucleus and promotes the expression of pro-inflammatory cytokines in a PKC-dependent manner after OGD/R. Sci. Rep..

[125] Tcherniuk S., Cenac N., Comte M., Frouard J., Errazuriz-Cerda E., Galabov A., Morange P.E., Vergnolle N., Si-Tahar M., Alessi M.C. (2016). Formyl Peptide Receptor 2 Plays a Deleterious Role During Influenza A Virus Infections. J. Infect. Dis..

[126] Foo S.L., Yap G., Cui J., Lim L.H.K. (2019). Annexin-A1—A Blessing or a Curse in Cancer?. Trends Mol. Med..

[127] Araujo T.G., Marangoni K., Rocha R.M., Maia Y.C., Araujo G.R., Alcantar T.M., Alves P.T., Calabria L., Neves A.F., Soares F.A. (2014). Dynamic dialog between cytokeratin 18 and annexin A1 in breast cancer: A transcriptional disequilibrium. Acta. Histochem..

[128] Gibbs L.D., Vishwanatha J.K. (2018). Prognostic impact of AnxA1 and AnxA2 gene expression in triple-negative breast cancer. Oncotarget.

[129] de Graauw M., van Miltenburg M.H., Schmidt M.K., Pont C., Lalai R., Kartopawiro J., Pardali E., Le Devedec S.E., Smit V.T., van der Wal A. (2010). Annexin A1 regulates TGF-beta signaling and promotes metastasis formation of basal-like breast cancer cells. Proc. Natl. Acad. Sci. USA.

[130] Vecchi L., Alves Pereira Zoia M., Goss Santos T., de Oliveira Beserra A., Colaco Ramos C.M., Franca Matias Colombo B., Paiva Maia Y.C., Piana de Andrade V., Teixeira Soares Mota S., Goncalves de Araujo T. (2018). Inhibition of the AnxA1/FPR1 autocrine axis reduces MDA-MB-231 breast cancer cell growth and aggressiveness in vitro and in vivo. Biochim. Biophys. Acta Mol. Cell Res..

[131] Mota S.T.S., Vecchi L., Alves D.A., Cordeiro A.O., Guimaraes G.S., Campos-Fernandez E., Maia Y.C.P., Dornelas B.C., Bezerra S.M., de Andrade V.P. (2020). Annexin A1 promotes the nuclear localization of the epidermal growth factor receptor in castration-resistant prostate cancer. Int. J. Biochem. Cell Biol..

[132] Boer J.C., Domanska U.M., Timmer-Bosscha H., Boer I.G., de Haas C.J., Joseph J.V., Kruyt F.A., de Vries E.G., den Dunnen W.F., van Strijp J.A. (2013). Inhibition of formyl peptide receptor in high-grade astrocytoma by CHemotaxis Inhibitory Protein of S. aureus. Br. J. Cancer.

[133] Locatelli I., Sutti S., Jindal A., Vacchiano M., Bozzola C., Reutelingsperger C., Kusters D., Bena S., Parola M., Paternostro C. (2014). Endogenous annexin A1 is a novel protective determinant in nonalcoholic steatohepatitis in mice. Hepatology.

[134] Moraes L.A., Kar S., Foo S.L., Gu T., Toh Y.Q., Ampomah P.B., Sachaphibulkij K., Yap G., Zharkova O., Lukman H.M. (2017). Annexin-A1 enhances breast cancer growth and migration by promoting alternative macrophage polarization in the tumour microenvironment. Sci. Rep..

[135] Ampomah P.B., Moraes L.A., Lukman H.M., Lim L.H.K. (2018). Formyl peptide receptor 2 is regulated by RNA mimics and viruses through an IFN-beta-STAT3-dependent pathway. FASEB J..

[136] Li Y., Cai L., Wang H., Wu P., Gu W., Chen Y., Hao H., Tang K., Yi P., Liu M. (2011). Pleiotropic regulation of macrophage polarization and tumorigenesis by formyl peptide receptor-2. Oncogene.

[137] Bai F., Zhang P., Fu Y., Chen H., Zhang M., Huang Q., Li D., Li B., Wu K. (2020). Targeting ANXA1 abrogates Treg-mediated immune suppression in triple-negative breast cancer. J. Immunother. Cancer.

[138] Oggero S., Austin-Williams S., Norling L.V. (2019). The Contrasting Role of Extracellular Vesicles in Vascular Inflammation and Tissue Repair. Front. Pharmacol..

[139] Kalluri R., LeBleu V.S. (2020). The biology, function, and biomedical applications of exosomes. Science.

[140] van Niel G., D’Angelo G., Raposo G. (2018). Shedding light on the cell biology of extracellular vesicles. Nat. Rev. Mol. Cell Biol..

[141] Sullivan R., Maresh G., Zhang X., Salomon C., Hooper J., Margolin D., Li L. (2017). The Emerging Roles of Extracellular Vesicles As Communication Vehicles within the Tumor Microenvironment and Beyond. Front. Endocrinol..

[142] Bian X., Xiao Y.T., Wu T., Yao M., Du L., Ren S., Wang J. (2019). Microvesicles and chemokines in tumor microenvironment: Mediators of intercellular communications in tumor progression. Mol. Cancer.

[143] Czystowska-Kuzmicz M., Whiteside T.L. (2021). The potential role of tumor-derived exosomes in diagnosis, prognosis, and response to therapy in cancer. Expert. Opin. Biol. Ther..

[144] Soldevilla B., Rodriguez M., San Millan C., Garcia V., Fernandez-Perianez R., Gil-Calderon B., Martin P., Garcia-Grande A., Silva J., Bonilla F. (2014). Tumor-derived exosomes are enriched in DeltaNp73, which promotes oncogenic potential in acceptor cells and correlates with patient survival. Hum. Mol. Genet..

[145] Ahmadi M., Rezaie J. (2020). Tumor cells derived-exosomes as angiogenenic agents: Possible therapeutic implications. J. Transl. Med..

[146] Peinado H., Aleckovic M., Lavotshkin S., Matei I., Costa-Silva B., Moreno-Bueno G., Hergueta-Redondo M., Williams C., Garcia-Santos G., Ghajar C. (2012). Melanoma exosomes educate bone marrow progenitor cells toward a pro-metastatic phenotype through MET. Nat. Med..

[147] Abusamra A.J., Zhong Z., Zheng X., Li M., Ichim T.E., Chin J.L., Min W.P. (2005). Tumor exosomes expressing Fas ligand mediate CD8+ T-cell apoptosis. Blood Cells Mol. Dis..

[148] Clayton A., Mason M.D. (2009). Exosomes in tumour immunity. Curr. Oncol..

[149] Clayton A., Mitchell J.P., Court J., Mason M.D., Tabi Z. (2007). Human tumor-derived exosomes selectively impair lymphocyte responses to interleukin-2. Cancer Res..

[150] Szczepanski M.J., Szajnik M., Welsh A., Whiteside T.L., Boyiadzis M. (2011). Blast-derived microvesicles in sera from patients with acute myeloid leukemia suppress natural killer cell function via membrane-associated transforming growth factor-beta1. Haematologica.

[151] Subra C., Grand D., Laulagnier K., Stella A., Lambeau G., Paillasse M., De Medina P., Monsarrat B., Perret B., Silvente-Poirot S. (2010). Exosomes account for vesicle-mediated transcellular transport of activatable phospholipases and prostaglandins. J. Lipid Res..

[152] Boilard E., Nigrovic P.A., Larabee K., Watts G.F., Coblyn J.S., Weinblatt M.E., Massarotti E.M., Remold-O’Donnell E., Farndale R.W., Ware J. (2010). Platelets amplify inflammation in arthritis via collagen-dependent microparticle production. Science.

[153] Boilard E. (2018). Extracellular vesicles and their content in bioactive lipid mediators: More than a sack of microRNA. J. Lipid Res..

[154] Prima V., Kaliberova L.N., Kaliberov S., Curiel D.T., Kusmartsev S. (2017). COX2/mPGES1/PGE2 pathway regulates PD-L1 expression in tumor-associated macrophages and myeloid-derived suppressor cells. Proc. Natl. Acad. Sci. USA.

[155] Xiang X., Poliakov A., Liu C., Liu Y., Deng Z.B., Wang J., Cheng Z., Shah S.V., Wang G.J., Zhang L. (2009). Induction of myeloid-derived suppressor cells by tumor exosomes. Int. J. Cancer.

[156] Kumar V., Patel S., Tcyganov E., Gabrilovich D.I. (2016). The Nature of Myeloid-Derived Suppressor Cells in the Tumor Microenvironment. Trends Immunol..

[157] Pessolano E., Belvedere R., Bizzarro V., Franco P., Marco I., Porta A., Tosco A., Parente L., Perretti M., Petrella A. (2018). Annexin A1 May Induce Pancreatic Cancer Progression as a Key Player of Extracellular Vesicles Effects as Evidenced in the In Vitro MIA PaCa-2 Model System. Int. J. Mol. Sci..

[158] Leoni G., Neumann P.A., Kamaly N., Quiros M., Nishio H., Jones H.R., Sumagin R., Hilgarth R.S., Alam A., Fredman G. (2015). Annexin A1-containing extracellular vesicles and polymeric nanoparticles promote epithelial wound repair. J. Clin. Investig..

[159] Jeppesen D.K., Fenix A.M., Franklin J.L., Higginbotham J.N., Zhang Q., Zimmerman L.J., Liebler D.C., Ping J., Liu Q., Evans R. (2019). Reassessment of Exosome Composition. Cell.

[160] Rogers M.A., Buffolo F., Schlotter F., Atkins S.K., Lee L.H., Halu A., Blaser M.C., Tsolaki E., Higashi H., Luther K. (2020). Annexin A1-dependent tethering promotes extracellular vesicle aggregation revealed with single-extracellular vesicle analysis. Sci. Adv..

[161] Draeger A., Wray S., Babiychuk E.B. (2005). Domain architecture of the smooth-muscle plasma membrane: Regulation by annexins. Biochem. J..

[162] Pessolano E., Belvedere R., Bizzarro V., Franco P., Marco I., Petrella F., Porta A., Tosco A., Parente L., Perretti M. (2019). Annexin A1 Contained in Extracellular Vesicles Promotes the Activation of Keratinocytes by Mesoglycan Effects: An Autocrine Loop Through FPRs. Cells.

[163] Aalberts M., Stout T.A., Stoorvogel W. (2014). Prostasomes: Extracellular vesicles from the prostate. Reproduction.

[164] Eden E.R., Sanchez-Heras E., Tsapara A., Sobota A., Levine T.P., Futter C.E. (2016). Annexin A1 Tethers Membrane Contact Sites that Mediate ER to Endosome Cholesterol Transport. Dev. Cell.

[165] Rentero C., Blanco-Munoz P., Meneses-Salas E., Grewal T., Enrich C. (2018). Annexins-Coordinators of Cholesterol Homeostasis in Endocytic Pathways. Int. J. Mol. Sci..

[166] Bardou M., Barkun A.N., Ghosn J., Hudson M., Rahme E. (2004). Effect of chronic intake of NSAIDs and cyclooxygenase 2-selective inhibitors on esophageal cancer incidence. Clin. Gastroenterol. Hepatol. Off. Clin. Pract. J. Am. Gastroenterol. Assoc..

[167] Rayburn E.R., Ezell S.J., Zhang R. (2009). Anti-Inflammatory Agents for Cancer Therapy. Mol. Cell. Pharmacol..

[168] de Gaetano G., Donati M.B., Cerletti C. (2003). Prevention of thrombosis and vascular inflammation: Benefits and limitations of selective or combined COX-1, COX-2 and 5-LOX inhibitors. Trends Pharmacol. Sci..

[169] Liaras K., Fesatidou M., Geronikaki A. (2018). Thiazoles and Thiazolidinones as COX/LOX Inhibitors. Molecules.

[170] Lin K.T., Wang L.H. (2016). New dimension of glucocorticoids in cancer treatment. Steroids.

[171] Yu J.A., Sadaria M.R., Meng X., Mitra S., Ao L., Fullerton D.A., Weyant M.J. (2012). Lung cancer cell invasion and expression of intercellular adhesion molecule-1 (ICAM-1) are attenuated by secretory phospholipase A(2) inhibition. J. Thorac. Cardiovasc. Surg..

[172] Makrilia N., Kollias A., Manolopoulos L., Syrigos K. (2009). Cell adhesion molecules: Role and clinical significance in cancer. Cancer Investig..

[173] Sadaria M.R., Meng X., Fullerton D.A., Reece T.B., Shah R.R., Grover F.L., Weyant M.J. (2011). Secretory phospholipase A2 inhibition attenuates intercellular adhesion molecule-1 expression in human esophageal adenocarcinoma cells. Ann. Thorac. Surg..

[174] Sadaria M.R., Yu J.A., Meng X., Fullerton D.A., Reece T.B., Weyant M.J. (2013). Secretory phospholipase A2 mediates human esophageal adenocarcinoma cell growth and proliferation via ERK 1/2 pathway. Anticancer Res..

[175] Nikolaou A., Kokotou M.G., Vasilakaki S., Kokotos G. (2019). Small-molecule inhibitors as potential therapeutics and as tools to understand the role of phospholipases A2. Biochim. Biophys. Acta BBA Mol. Cell Biol. Lipids.

[176] Rosenson R.S., Hislop C., Elliott M., Stasiv Y., Goulder M., Waters D. (2010). Effects of varespladib methyl on biomarkers and major cardiovascular events in acute coronary syndrome patients. J. Am. Coll. Cardiol..

[177] Suckling K. (2010). Phospholipase A2s: Developing drug targets for atherosclerosis. Atherosclerosis.

[178] Fraser H., Hislop C., Christie R.M., Rick H.L., Reidy C.A., Chouinard M.L., Eacho P.I., Gould K.E., Trias J. (2009). Varespladib (A-002), a secretory phospholipase A2 inhibitor, reduces atherosclerosis and aneurysm formation in ApoE-/- mice. J. Cardiovasc. Pharmacol..

[179] Nicholls S.J., Kastelein J.J., Schwartz G.G., Bash D., Rosenson R.S., Cavender M.A., Brennan D.M., Koenig W., Jukema J.W., Nambi V. (2014). Varespladib and cardiovascular events in patients with an acute coronary syndrome: The VISTA-16 randomized clinical trial. Jama.

[180] Dong Z., Liu Y., Scott K.F., Levin L., Gaitonde K., Bracken R.B., Burke B., Zhai Q.J., Wang J., Oleksowicz L. (2010). Secretory phospholipase A2-IIa is involved in prostate cancer progression and may potentially serve as a biomarker for prostate cancer. Carcinogenesis.

[181] Moon T.C., Hwang H.S., Quan Z., Son K.H., Kim C.H., Kim H.P., Kang S.S., Son J.K., Chang H.W. (2006). Ochnaflavone, naturally occurring biflavonoid, inhibits phospholipase A2 dependent phosphatidylethanolamine degradation in a CCl4-induced rat liver microsome. Biol. Pharm. Bull..

[182] Suh S.J., Jin U.H., Kim S.H., Chang H.W., Son J.K., Lee S.H., Son K.H., Kim C.H. (2006). Ochnaflavone inhibits TNF-alpha-induced human VSMC proliferation via regulation of cell cycle, ERK1/2, and MMP-9. J. Cell. Biochem..

[183] Suh S.J., Chung T.W., Son M.J., Kim S.H., Moon T.C., Son K.H., Kim H.P., Chang H.W., Kim C.H. (2006). The naturally occurring biflavonoid, ochnaflavone, inhibits LPS-induced iNOS expression, which is mediated by ERK1/2 via NF-kappaB regulation, in RAW264.7 cells. Arch. Biochem. Biophys..

[184] Elhady S.S., El-Halawany A.M., Alahdal A.M., Hassanean H.A., Ahmed S.A. (2016). A New Bioactive Metabolite Isolated from the Red Sea Marine Sponge Hyrtios erectus. Molecules.

[185] Yap W.H., Ahmed N., Lim Y.M. (2016). Inhibition of Human Group IIA-Secreted Phospholipase A2 and THP-1 Monocyte Recruitment by Maslinic Acid. Lipids.

[186] Yap W.H., Ooi B.K., Ahmed N., Lim Y.M. (2018). Maslinic acid modulates secreted phospholipase A2-IIA (sPLA2-IIA)-mediated inflammatory effects in macrophage foam cells formation. J. Biosci..

[187] Wei Q., Zhang B., Li P., Wen X., Yang J. (2019). Maslinic Acid Inhibits Colon Tumorigenesis by the AMPK-mTOR Signaling Pathway. J. Agric. Food Chem..

[188] Jain R., Grover A. (2020). Maslinic acid differentially exploits the MAPK pathway in estrogen-positive and triple-negative breast cancer to induce mitochondrion-mediated, caspase-independent apoptosis. Apoptosis.

[189] Gimenes S.N.C., Lopes D.S., Alves P.T., Azevedo F., Vecchi L., Goulart L.R., Rodrigues T.C.S., Santos A.L.Q., Brites V.L.C., Teixeira T.L. (2017). Antitumoral effects of gammaCdcPLI, a PLA2 inhibitor from Crotalus durissus collilineatus via PI3K/Akt pathway on MDA-MB-231 breast cancer cell. Sci. Rep..

[190] Zhang M., Wu J.S., Yang X., Pang X., Li L., Wang S.S., Wu J.B., Tang Y.J., Liang X.H., Zheng M. (2018). Overexpression Cathepsin D Contributes to Perineural Invasion of Salivary Adenoid Cystic Carcinoma. Front. Oncol..

[191] Zoia M.A.P., Azevedo F.V.P., Vecchi L., Mota S.T.S., Rodovalho V.R., Cordeiro A.O., Correia L.I.V., Silva A.C.A., Avila V.M.R., Araujo T.G. (2019). Inhibition of Triple-Negative Breast Cancer Cell Aggressiveness by Cathepsin D Blockage: Role of Annexin A1. Int. J. Mol. Sci..

